# A highly sensitive bead-based flow cytometric competitive binding assay to detect SARS-CoV-2 neutralizing antibody activity

**DOI:** 10.3389/fimmu.2022.1041860

**Published:** 2022-11-30

**Authors:** Xiangyu Yao, Zhichao Zhang, Qingmin Mei, Shenwei Li, Li Xing, Yali Long, Demei Zhang, Jing Wang, Xiedong Wang, Bin Xie, Bo Yang, Yong Gao, Changxin Wu, Qinglai Meng

**Affiliations:** ^1^ The Key Laboratory of Chemical Biology and Molecular Engineering of National Ministry of Education, Shanxi Provincial Key Laboratory of Medical Molecular Cell Biology, Institute of Biomedical Sciences, Shanxi University, Taiyuan, China; ^2^ Shanxi Academy of Advanced Research and Innovation, Taiyuan, China; ^3^ The First Affiliated Hospital of USTC, Division of Life Sciences and Medicine, University of Science and Technology of China (USTC), Hefei, China; ^4^ The Support Department for Health and Quarantine, Shanghai International Travel Healthcare Center (Shanghai Customs Port Clinic), Shanghai, China; ^5^ Shanxi Provincial Key Laboratory for Major Infectious Disease Response, Taiyuan, China; ^6^ Hospital of Shanxi University, Shanxi University, Taiyuan, China; ^7^ Taiyuan Blood Center, Taiyuan, China; ^8^ Pure and Applied Biochemistry, Department of Chemistry, Lund University, Lund, Sweden

**Keywords:** SARS-CoV-2, neutralizing antibody, neutralization test, competitive binding, bead based, omicron

## Abstract

Accurate detection of SARS-CoV-2 neutralizing antibody (nAb) is critical for assessing the immunity levels after virus infection or vaccination. As fast, cost-effective alternatives to viral infection-based assays, competitive binding (CB) assays were developed to quantitate nAb by monitoring the ability of sera to inhibit the binding of viral spike (S) protein to the angiotensin converting enzyme 2 (ACE2) receptor. Herein, we established a bead-based flow cytometric CB assay and tested the detection performance of six combination models, i.e. immobilized ACE2 and soluble Fc-tagged S1 subunit of S protein (iACE2/S1-Fc), immobilized ACE2 and soluble Fc-tagged receptor binding domain (RBD) of S protein (iACE2/RBD-Fc), immobilized S1 and soluble Fc-tagged ACE2 (iS1/ACE2-Fc), immobilized S1 and soluble His-tagged ACE2 (iS1/ACE2-His), immobilized RBD and soluble Fc-tagged ACE2 (iRBD/ACE2-Fc), and immobilized RBD and soluble His-tagged ACE2 (iRBD/ACE2-His). Using SARS-CoV-2 monoclonal antibodies and sera of convalescent COVID-19 patients and vaccinated subjects, the combination models iACE2/RBD-Fc, iACE2/S1-Fc and iS1/ACE2-His were identified to be able to specifically detect SARS-CoV-2 nAb, among which iACE2/RBD-Fc model showed the highest sensitivity, superior to a commercial SARS-CoV-2 surrogate virus neutralization test (sVNT) ELISA kit. Further studies demonstrated that the sensitivity and specificity of CB assays were affected by the tag of ACE2, type of spike and method of measuring binding rate between ACE2 and spike. Moreover, the iACE2/RBD-Fc model showed good performance in detecting kinetic development of nAb against both the prototype SARS-CoV-2 strain and an omicron variant of SARS-CoV-2 in people immunized by an inactivated SARS-CoV-2 vaccine, and the results of iACE2/RBD-Fc model are correlated well with those of live virus-based and pseudovirus-based neutralization tests, demonstrating the potential to be developed into a highly sensitive, specific, versatile and high-throughput method for detecting SARS-CoV-2 nAb in clinical practice.

## Introduction

Coronavirus disease 2019 (COVID-19) pandemic was caused by severe acute respiratory syndrome coronavirus 2 (SARS-CoV-2), leading to a tremendous loss of human lives and economy. SARS-CoV-2 initiates infection by binding of the viral spike (S) protein to the cellular receptor angiotensin converting enzyme 2 (ACE2) (1). The S protein is composed of S1 and S2 subunits. The S1 subunit, in particular the receptor binding domain (RBD) of S1, is responsible for the binding of S protein to ACE2, while S2 for the virus-cell membrane fusion (2, 3). RBD is a major inducer for the generation of SARS-CoV-2 specific neutralizing antibody (nAb) to block viral infection (4–7). Due to the pivotal role in prevention and elimination of SARS-CoV-2 infection, nAb becomes a focus of SARS-CoV-2 drug development and the level of nAb in human body serves as the most clinically relevant parameter for assessing efficacy of SARS-CoV-2 vaccines (8–11).

So far, multiple assays have been developed to assess activity of SARS-CoV-2 nAb (12–19). Plaque reduction neutralization test (PRNT) was regarded as a golden standard assay, because it assesses the effect of nAb on authentic live SARS-CoV-2 infection of permissive cells. However, the 7 days turnaround time, biosafety level III (BL3) performance condition, and low throughput data production profoundly limit its application (12). Pseudovirus based neutralization test (PNT) was regarded as another standard assay to assess the activity of nAb induced by a variety of vaccines against the prototype SARS-CoV-2 or emerging variants (12, 13). The 24-48 hours turnaround time, biosafety level 2 (BL2) facilities and low automation still restrict the application of PNT in large population based clinical tests.

To overcome the limits of the aforementioned viral infection-based nAb assays, a strategy based on competitive binding (CB) to S1 or RBD of S protein between SARS-CoV-2 nAb and human ACE2 was established recently (14–20). The CB-based nAb assays usually have a 1-2 hours turnaround time, need less amounts of recombinant proteins, and can be converted into high throughput detection methods by combining with a magnetic bead based chemiluminescent or multiplex immunoassay (14, 16, 18). Moreover, the results of CB based nAb assays demonstrated excellent correlation with that of PRNT and PNT in assessment of nAb titer against an authentic prototype SARS-CoV-2 or emerging viral variants including delta and omicron (14, 16, 21).

In the CB-based nAb assays, either ACE2 protein (14, 19) or RBD, S1 subunit of S protein, or even the whole S protein (15–18, 20) was immobilized to catch the corresponding soluble counter-partner protein (14–19). However, the detection performance of these different immobilization strategies was never comparatively analyzed. In this study, we used magnetic beads to establish a flow cytometric CB-based SARS-CoV-2 nAb assay to systemically address this question and found a highly sensitive and specific CB assay model.

## Material and methods

### Recombinant proteins

All the recombinant proteins used in this study were produced from HEK293 cells. Biotinylated His- Avi-tagged human ACE2 (ACE2 -Avi) and RBD of SARS-CoV-2 S protein (RBD-Avi, Wuhan strain) were purchased from Kactus Biosystem (Shanghai, China). Mouse IgG1 Fc-tagged SARS-CoV-2 S1 (Wuhan strain), RBD (Wuhan strain and Omicron BA.1 variant), and human ACE2 (S1-Fc, RBD-Fc and ACE2-Fc, respectively) were purchased from Sino Biological (Beijing, China). Biotinylated His- Avi-tagged SARS-CoV-2 S1 (S1-Avi, Wuhan strain) and His-tagged recombinant human ACE2 (ACE2-His) were purchased from Acro biosystems (Beijing, China). The protein sequences of spike RBD or S1 protein are identical to that of SARS-CoV-2 Wuhan strain (GenBank accession No. QHD43416.1) or its omicron BA.1 variant with mutations of G339D, S371L, S373P, S375F, K417N, N440K, G446S, S477N, T478K, E484A, Q493R, G496S, Q498R, N501Y, Y505H. For the convenience, the RBD-Fc with the RBD sequence of Wuhan strain was represented as the RBD-Fc otherwise as the RBD-Fc Wuhan for differentiation from the RBD-Fc with RBD sequence of omicron BA.1 variant, which is represented as the RBD-Fc omicron.

### Antibodies

Recombinant human IgG1 isotype control antibody (Clone. No. QA16A12), mouse IgG1 isotype control antibody (Clone. No. MOPC-21), PE-conjugated mouse anti His tag mAb (Clone. No. J095G46) and PE-conjugated isotype control antibody (Clone. No. MOPC-173) were purchased from Biolegend (San Diego, CA). Other antibodies used in this study include AF488-conjugated goat anti human or mouse IgG (H+L) polyclonal antibodies (pAbs) (Thermo Fisher, Rockford, IL), rabbit anti spike RBD neutralizing mAb 001 (Sino Biological, Beijing, China), recombinant human IgG1 anti spike RBD neutralizing mAb BDAB0065 (Biodragon, Suzhou, China), anti-spike RBD non-neutralizing mAb HMB001-N (Bioworld Technology, Nanjing, China) and recombinant human IgG1 anti SARS spike RBD neutralizing mAb CR3022 (Absolute Antibody, Cleveland, UK). According to the technical datasheets, the 001 mAb is a potent strain-specific nAb against Wuhan strain other than any omicron variants, whereas the BDAB0065 mAb is a broadly nAb against both Wuhan strain and omicron BA.1. The CR3022 mAb has a strong cross binding activity to SARS-CoV-2 RBD protein, but doesn’t show neutralizing activity to SARS-CoV-2 (22).

### Immobilization of proteins to magnetic beads

Streptavidin (SA) beads (Dynabeads MyOne Streptavdin T1, Thermo Fisher, Baltics, UAB) were used to immobilize biotinylated recombinant ACE2, spike RBD or S1 protein according to the instructions of manufacturer. A DynaMag-96 Side Skirt (Thermo Fisher) was used to separate magnetic beads for washing and changing buffer. Briefly, 400 ng of ACE2-Avi or S1-Avi or 200 ng of RBD-Avi were mixed with 10 μg (1μl) of SA bead suspension in 20 μl of PBS (pH7.4) for 40 minutes at room temperature (RT). The final concentrations of ACE2 -Avi, S1-Avi and RBD-Avi in the reaction mixtures were 20, 20 and 10 μg/ml, respectively. After washing once, the protein-immobilized SA beads were suspended with 80 μl of PBS containing 0.1% BSA and then stored at 4°C. The ACE2-Avi-, S1-Avi- and RBD-Avi-immobilized SA beads were referred to as ACE2 bead, S1 bead and RBD bead, respectively.

### Sample collection

To assess performance of the iACE2/RBD-Fc model to detect nAb induced by a SARS-CoV-2 vaccine in the setting of real-world, sixty healthy people were enrolled in Shanxi University and vaccinated by an inactivated SARS-CoV-2 vaccine (BBIBP-CorV, Beijing Institute of Biological Product Co., Ltd. Beijing, China) for three times. The interval time between the 1^st^ and the 2^nd^ dose of immunization and between the 2^nd^ and the 3^rd^ dose of immunization were 4 weeks and 6 months, respectively. Serum samples were collected from all sixty vaccinees at 1-2 days before the 1^st^ dose of immunization, 2 weeks post the 2^nd^ dose and the 3^rd^ dose of immunization, or from twenty-five, twenty-eight and thirty-four vaccinees at 4 and 6 months post the 2^nd^ dose of immunization and 2 weeks post the 3^rd^ dose of immunization, respectively. 28 plasma samples from convalescent COVID-19 patients previously infected by Wuhan strain of SARS-CoV-2 were collected in Taiyuan blood center at approximate 2 weeks post discharge from Taiyuan Fourth People’s Hospital. Among those, panel A including 16 plasma samples were used for correlation analysis of nAb activity determined by the PNT and the iACE2/RBD-Fc model, and panel B including 12 plasma samples were totally or partially used for sensitivity comparison between assays and mechanism investigation.

For correlation analysis of nAb activity determined by the PRNT and the iACE2/RBD-Fc assay, 12 convalescent COVID-19 patient plasma samples and 8 SARS-CoV-2 vaccine serum samples that were randomly selected from a panel of 20 convalescent patient plasma samples (23) and 62 vaccine serum samples (unpublished data) were used. The samples were collected in the first affiliated hospital of University of Science and Technology of China (USTC) from convalescent patients at 3–5 months after the initiation of the disease or from vaccinees at 2 weeks post the 2^nd^ dose of an inactivated SARS-CoV-2 vaccine (BBIBP-CorV). To assess efficacy of the iACE2/RBD-Fc assay to detect SARS-CoV-2 omicron strain specific nAb, plasma samples collected in the first affiliated hospital of USTC from 10 vaccine (BBIBP-CorV) recipients undergoing breakthrough infection by an omicron BA.1 variant were used. The omicron BA.1 breakthrough infection was determined by RT-PCR and the samples were collected within one to two weeks post breakthrough infection. The BBIBP CorV vaccine were confirmed to be able to efficiently induce the production of nAb against SARS-CoV-2 Wuhan strain as well as multiple variants after a booster vaccination (24). All plasma and serum samples were aliquoted and stored at -80°C until usage. The informed consent was obtained from the participants enrolled in Shanxi University and the first affiliated hospital of USTC, or waivered from the convalescent COVID-19 patients and normal healthy donors enrolled in Taiyuan blood. The study was approved by the ethical committee of Shanxi University (No. SXULL2021048), Taiyuan blood center (No. 2020-2) and the first affiliated hospital of USTC (No. 2020-SZ(H)-016).

### Bead-based flow cytometric SARS-CoV-2 virus neutralization test (BFNT)

**Figure 1:**
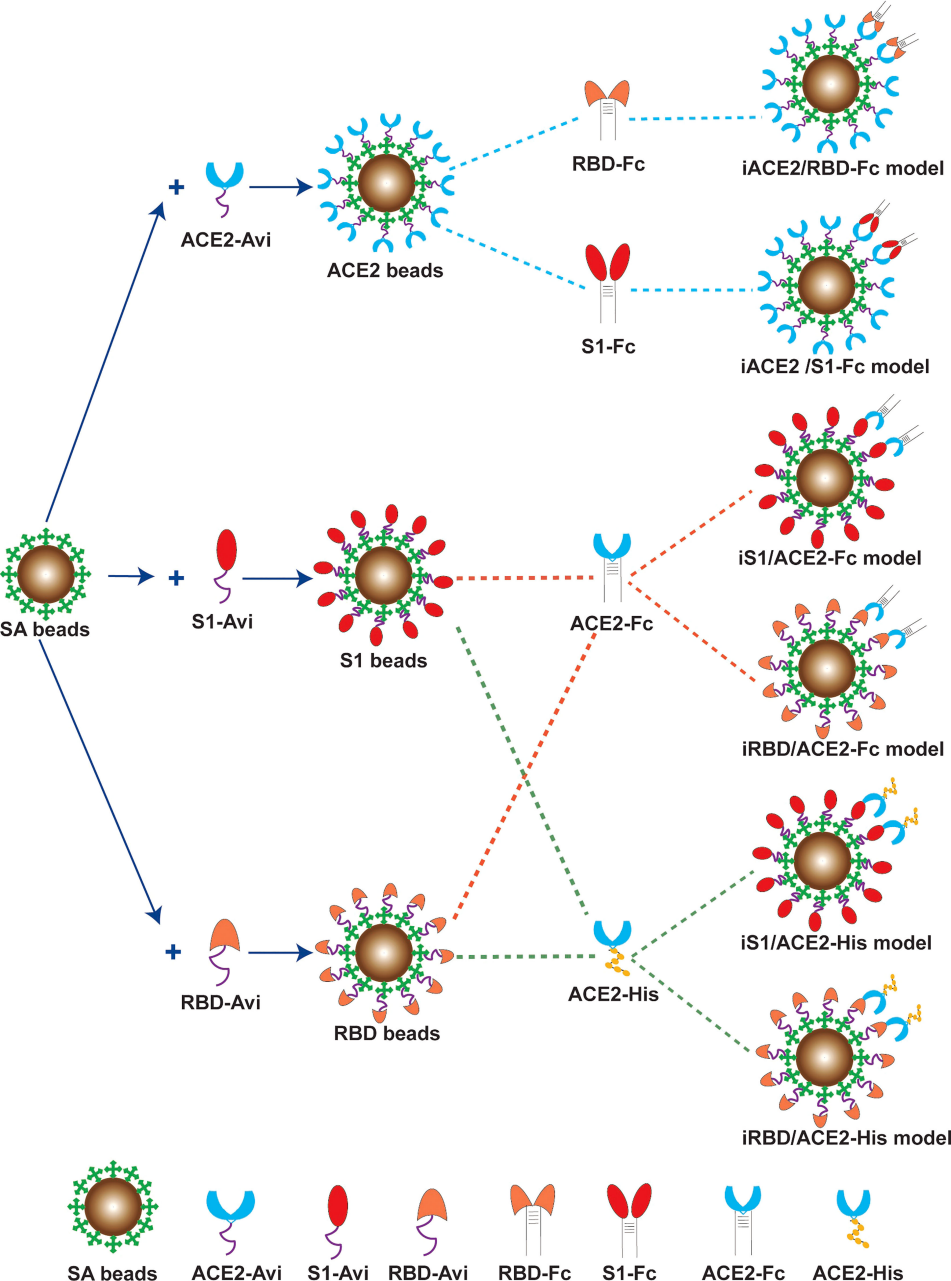
Schematic diagram of the six BFNT models to detect SARS-CoV-2 nAb. Streptavdin coated magnetic bead (SA bead) was immobilized by recombinant human ACE2 protein with a biotinylated Avi tag (ACE2-Avi), recombinant SARS-CoV-2 Spike S1 protein with a biotinylated Avi tag (S1-Avi) and recombinant SARS-CoV-2 spike RBD protein with a biotinylated Avi tag (RBD-Avi) to generate ACE2 bead, S1 bead and RBD bead, respectively. The ACE2 bead with a soluble RBD-Fc or S1-Fc protein were combined to establish iACE2/RBD-Fc or iACE2/S1-Fc model. The RBD bead or the S1 bead was combined with a ACE2-Fc or ACE2-His protein to establish iRBD/ACE2-Fc, iS1/ACE2-Fc, iRBD/ACE2-His or iS1/ACE2-His model.

As depicted in **Figure 1**, six combination models for the competitive binding-based nAb assay were developed to assess SARS-CoV-2 nAb activity, *i.e.* immobilized ACE2 versus soluble Fc-tagged S1 of S protein (iACE2/S1-Fc), immobilized ACE2 versus soluble Fc-tagged RBD of S protein (iACE2/RBD-Fc), immobilized S1 versus soluble Fc-tagged ACE2 (iS1/ACE2-Fc), immobilized S1 versus soluble His-tagged ACE2 (iS1/ACE2-His), immobilized RBD versus soluble Fc-tagged ACE2 (iRBD/ACE2-Fc), and immobilized RBD versus soluble His-tagged ACE2 (iRBD/ACE2-His). 0.1% BSA (pH7.4) PBS and 0.02% Tween-20 PBS (pH7.4) were used as dilution solution and washing solution, respectively.

To determine nAb activity using the iACE2/RBD-Fc or iACE2/S1-Fc models, serum, plasma, spike mAb or negative control antibody (human IgG1) were diluted, added into wells of a U-bottom 96 well plate in duplicate, and then pre-incubated with 2 ng/ml of soluble RBD-Fc Wuhan, RBD-Fc omicron or mouse IgG1 (as an isotype control of staining) for the iACE2/RBD-Fc model or 40 ng/ml of soluble S1-Fc or mouse IgG1 for the iACE2/S1-Fc model in 100 μl of dilution solution at 37°C for 30 mins, followed by co-incubation with 1 μl of ACE2 beads at RT for further 30 mins. After twice washing, the beads in each well were stained by 0.5 μg/ml of AF488-conjugated goat anti mouse IgG polyclonal antibody (pAb) at RT for 20 mins. After washing twice, the beads were suspended in washing solution and then subjected to flow cytometry analysis (Fortessa, BD) to quantitate RBD-Fc- or S1-Fc-bound ACE2 beads. The mouse IgG1-treated beads were used to set up a gate for identification of RBD-Fc or S1-Fc positive ACE2 beads.

To determine nAb activity using iRBD/ACE2-Fc, iRBD/ACE2-His, iS1/ACE2-Fc or iS1/ACE2-His model, serum, plasma, spike mAb or negative control antibody (human IgG1) was diluted to 2 times of the anticipated final concentrations, added into wells of a U-bottom 96 well plate in duplicate, and then pre-incubated at 37°C for 30 mins with the same volume of RBD bead or S1 bead solution diluted from 1 μl of the bead stock, followed by co-incubation with 16 ng/ml of soluble ACE2-Fc (for iRBD/ACE2-Fc model), 64 ng/ml of soluble ACE2-Fc (for iS1/ACE2-Fc model) or 400 ng/ml of soluble ACE2-His (for iRBD/ACE2-His and iS1/ACE2-His models) at RT for further 30 mins. After twice washing, the beads in each well were stained by 0.5 μg/ml of AF488-conjugated goat anti mouse IgG pAb (for iRBD/ACE2-Fc and iS1/ACE2-Fc models) or 0.12 μg/ml of PE-conjugated anti His tag mAb (for iRBD/ACE2-His and iS1/ACE2-His models) at RT for 20 mins. After washing twice, the beads were suspended in washing solution and then subjected to flow cytometry analysis to quantitate ACE2-his or ACE2-Fc bound RBD or S1 beads. The beads without treatment with soluble ACE2-His or ACE2-mFc were used to set up a gate for identification of ACE2-His or ACE2-Fc positive RBD or S1 beads. The results were analyzed using FlowJo (version 10).

The efficacy of nAb-mediated binding inhibition between the soluble protein and its immobilized counterpart protein under each diluted sample was determined by measuring the percentage of soluble protein bound beads and calculated as [(% maximum binding between the soluble protein and its counterpart protein-immobilized bead - % soluble protein bound beads in the presence of nAb or serum in the diluted sample)/% maximum binding between the soluble protein and its counterpart protein-immobilized bead] x 100 or by using mean fluorescence intensity (MFI) of the bound soluble protein on the whole population of its counterpart protein-immobilized beads and calculated as [(MFI of maximum binding between the soluble protein and its counterpart protein-immobilized bead – MFI of the bound soluble protein on the whole population of its counterpart protein-immobilized beads in the presence of nAb or serum in the diluted sample)/MFI of maximum binding between the soluble protein and its counterpart protein-immobilized bead] x 100. The value of percentage or MFI of maximum binding between the soluble protein and its counterpart protein-immobilized bead was determined using the samples treated with negative control antibody (human IgG1).

### ELISA based SARS-CoV-2 surrogate virus neutralization test (sVNT)

The ID50 (50% of maximal inhibitory dose) of samples were determined using an ELISA based sVNT kit (GenScript cPass SARS-CoV-2 neutralization Antibody detection kit, GenScript, China) according to instruction of the manufacturer with a minor modification. Briefly, the sample was 3-fold serially diluted with the dilution buffer from 1:2.5 to 607.5 for the vaccinated serum samples or from 1:15 to 1:3645 for the convalescent patient plasma samples. The negative and positive control samples of the sVNT kit were 10-fold diluted with the dilution buffer. The diluted samples were mixed with the equal volume of the diluted HRP-conjugated RBD and incubated at 37 °C for 30 mins, followed by transferring to ACE2 protein-precoated wells in a 96 well plate and then incubating at 37 °C for a further 15 mins. After four times washing, each well was added with TMB solution, incubated at RT for 15 mins, and then added with stop solution. The plate was read at 450 nm immediately. The percent binding inhibition between the HRP-RBD and ACE2 protein was calculated as (1-OD value of the diluted sample/OD value of the diluted negative control) x 100.

### Pseudovirus neutralization test (PNT)

In this study, the VSV-based SARS-CoV-2 PNT and the lentivirus-based SARS-CoV-2 PNT were employed to determine nAb activity against two SARS-CoV-2 pseudoviruses bearing spike protein of the Wuhan strain and the omicron BA.1 variant, respectively. The VSV-based SARS-CoV-2 PNT was performed as previously described (13). Briefly, Wuhan strain pseudovirus were prepared by subsequent transfection and infection of 293T cells with a SARS-CoV-2 S protein- expressing plasmid and a VSV G pseudovirus (G*ΔG-VSV), respectively. At 24 and 48 hours post infection/transfection, cell supernatant containing the pseudovirus was harvested, titrated, and stored at -80 °C until usage. The heat-inactivated convalescent patient plasma was 3-fold serially diluted with DMEM complete medium from 1:15 to 1:3645, transferred to wells of 96 well plate in duplicate, incubated with the same volume of pseudovirus solution containing 650 TCID50 of SARS-CoV-2 pesudovirus at 37 °C for 1 hour, and then mixed with 2 x 10^4^ Vero cells. After culturing at 37 °C with 5% CO2 for 24 hours, the cells in each well were lysed, treated with luciferase substrate, and then the luminescence was measured by a luminescent reader (Perkin Elmer).

The lentiviral vector-based SARS-CoV-2 PNT was performed as previously described (23). Briefly, omicron BA.1 variant pseudovirus were prepared by co-transfection of HEK 293T cells with a lentiviral packaging plasmid pNL4-3 Luc+R-E- and a plasmid encoding spike protein of the omicron BA.1. At 48 hours post transfection, cell supernatant containing the pseudovirus was collected, centrifuged, filtered, and stored at -80 °C until usage. HEK293T/hACE2 cells that stably express human ACE2 were seeded in 96-well cell plates at 10,000 cells/well and were cultured for 24 h before detection. The plasma samples were 2-fold serially diluted in the culture medium with the initial dilution of 1:20, mixed with 50 μl of pseudovirus with the values of relative luminescence unit (RLU) at approximately 1.0×10^5^, then incubated at 37°C for 1 h. Afterward, the mixtures of the diluted plasma and pseudovirus were added to HEK293T/hACE2 cells and further incubated at 37°C with 5% CO2 for 48 h. The values of RLU of cell culture well were measured by the Britelite plus Reporter Gene Assay System (PerkinElmer). For both PNTs, the inhibition rate was calculated by comparing the OD value to the cell well treated by the pseudovirus solution alone.

### Plaque reduction neutralization test (PRNT)

Plaque reduction neutralization test (PRNT) using an authentic live SARS-CoV-2 virus was performed according to the previous description with minor modifications (25). Briefly, Vero E6 cells were seeded in 96-well plates and incubated at 37°C overnight. Two-fold dilution of the convalescent plasma with 1:20 as the initial dilution fold or the vaccine serum with 1:2 as the initial dilution fold were mixed with the same volume of SARS-CoV-2 authentic virus at 100 TCID50 and incubated for 1 h at 37°C. The mixtures of diluted plasma or serum and virus were then transferred to Vero E6 cell wells and incubated at 37°C for 1 h. Afterward, the supernatant was replaced with the DMEM maintaining culture media and the cells were further cultured for 5 days at 37°C, cytopathic effect (CPE) caused by the infection was recorded using optical microscopy. All experiments with the authentic virus, which was isolated from a COVID-19 patient in Anhui province, were conducted in Biosafety Level 3 (BSL-3) laboratory in Anhui Provincial Center for Disease Control and Prevention. All experiments were complied with the biosecurity and institutional safety. The percent plaque reduction was calculated as (1- plaque number in cell well treated by mixture of the diluted sample and the authentic virus/plaque number in cell well treated by the authentic virus alone) x 100.

### Statistical analysis

Statistical analyses were performed using GraphPad Prism 7.0. The paired and unpaired Student *t* test were used to compare differences, and a 2-tailed P value <0.05 was considered significant. Receiver operating characteristic (ROC) curve analysis was used to determine sensitivity and specificity of the iACE2/RBD-Fc assay. Spearman’s correlation coefficient was used to determine the correlation between different experiments. Nonlinear four parameter curve fitting analysis of the log (agonist) verse response was used to generate serum or plasma dilution response inhibition curves from which 50% of maximal inhibitory dose (ID50) of the tested sample was determined.

## Results

### Optimal immobilization of ACE2, S1, or RBD protein on SA beads

We first optimized the conditions for immobilizing biotinylated ACE2 or RBD, S1 protein on SA beads. The immobilized ACE2-Avi was evaluated by detecting the binding of soluble RBD-Fc after treatment with RBD-Fc at a saturating concentration. As shown in **Figure S1A**, the MFI of RBD-Fc on the ACE2 bead prepared at 20 μg/ml ACE2 protein approached to the maximum, indicating that SA beads were nearly maximally immobilized by the ACE2-Avi protein at 20 μg/ml concentration. The immobilized biotinylated RBD-Avi or S1-Avi protein was evaluated by indirect antibody staining of RBD or S1 protein -immobilized beads. As shown in **Figures S1B, C**, the MFIs of RBD-Avi or S1-Avi protein on the RBD or S1 beads prepared at 10μg/ml of RBD-Avi or S1-Avi protein reached the maximum, indicating that 10 μg/ml concentration of RBD or S1 protein is optimal for immobilization of both proteins. Therefore, in this study the ACE2 bead, RBD bead and S1 bead were prepared with ACE2 protein, RBD protein, and S1 protein at 20, 10 and 10 μg/ml, respectively.

### Binding of ACE2 bead, RBD bead and S1 bead with their counterpart soluble proteins

Next, we examined the dose dependent binding of soluble RBD-Fc and S1-Fc to ACE2 bead or soluble ACE2-Fc and ACE-His to RDB bead or S1 bead to determine the optimal binding concentrations of the tested soluble proteins. A threshold concentration at which the binding of the tested soluble protein to its counterpart protein-immobilized bead reached a plateau was deemed as the optimal binding concentration of the tested protein. As shown in **Supplementary Figure 2**, the binding of soluble RBD-Fc and S1-Fc to ACE2 bead reached a plateau level (≥ 90%) at concentrations of 2 ng/ml and 32 ng/ml, respectively (**Supplementary Figures 2A, B**), while the binding of soluble ACE2-Fc to RBD bead and S1 bead reached a plateau level at concentrations of 16 ng/ml and 64 ng/ml, respectively (**Supplementary Figures 2C, D**). The binding of soluble ACE2-His to RBD bead or S1 bead reached a plateau level at concentration of 400 ng/ml (**Supplementary Figures 2E, F**). On the other hand, the threshold concentration of soluble RBD-Fc omicron binding on ACE2 bead in a plateau level was 2 ng/ml (**Supplementary Figure 2G**). Hence, 2 ng/ml and 40 ng/ml were determined as the optimal binding concentrations of soluble RBD-Fc (for both Wuhan strain and omicron variant) and S1-Fc for iACE2/RBD-Fc and iACE2/S1-Fc combination models, respectively; 16 ng/ml and 64 ng/ml were determined as the optimal binding concentrations of ACE2-Fc for iRBD/ACE2-Fc and iS1/ACE2-Fc models, respectively. 400 ng/ml was the optimal binding concentration of soluble ACE2-His for iRBD/sACE2-His and iS1/sACE2-His combination models.

### Evaluation of six BFNT models using SARS-CoV-2 spike RBD mAbs

**Figure 2:**
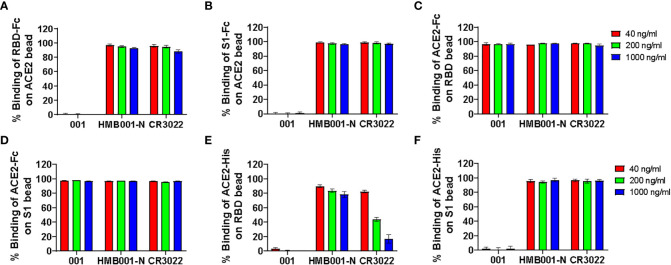
Detection of three function-known spike RBD antibodies by the six BFNT models. Two SARS-CoV-2 non-neutralizing mAbs (HMB001-N and CR3022) and one SARS-CoV-2 neutralizing mAb (001) were employed to assess specificity and sensitivity of the six BFNT models. Efficacies of binding inhibition of the RBD-Fc on the ACE2 bead, the S1-Fc on the ACE2 bead, the ACE2-Fc on RBD bead, the ACE2-Fc on the S1 bead, the ACE2-His on the RBD bead and the ACE2-His on the S1 bead by the three mAbs at concentrations of 40, 200 and 1000 ng/ml were shown in **(A)** iACE2/RBD-Fc model, **(B)** iACE2/S1-Fc model, **(C)** iRBD/ACE2-Fc model, **(D)** iS1/ACE2-Fc model, **(E)** iRBD/ACE2-His model and **(F)** iS1/ACE2-His model, respectively.

Three SARS-CoV-2 RBD specific mAbs were employed to evaluate the sensitivity and specificity of six BFNT combination models in detection of SARS-CoV-2 nAb. The specificity was assessed using one non-neutralizing mAb (HMB001-N) and one SARS neutralizing but SARS-CoV-2 non-neutralizing mAb (CR3022) (22), and the sensitivity was assessed by one SARS-CoV-2 neutralizing mAb (001). As shown in **Figures 2A–F**, neutralizing mAb 001-mediated inhibition of binding between ACE2 and RDB or S1 protein were observed at all tested concentrations for the models iACE2/RBD-Fc, iACE2/S1-Fc, iRBD/ACE2-His and iS1/ACE2-His, but not for models iRBD/ACE2-Fc and iS1/ACE2-Fc, indicating the latter two models have the poor sensitivity. On the other hand, two non-neutralizing mAbs HMB001-N and CR3022-mediated dose dependent inhibition of binding between ACE2 and RDB or S1 protein was observed for iRBD/ACE2-His model (**Figure 2E**), indicating the lack of specificity for this model. Overall, the results show that models iACE2/RBD-Fc, iACE2/S1-Fc and iS1/ACE2-His have both sufficient sensitivity and specificity. Therefore, these three models were further evaluated.

### Comparing the detection sensitivity of models iACE2/RBD-Fc, iACE2/S1-Fc and iS1/ACE2-His using plasma and serum samples

**Figure 3:**
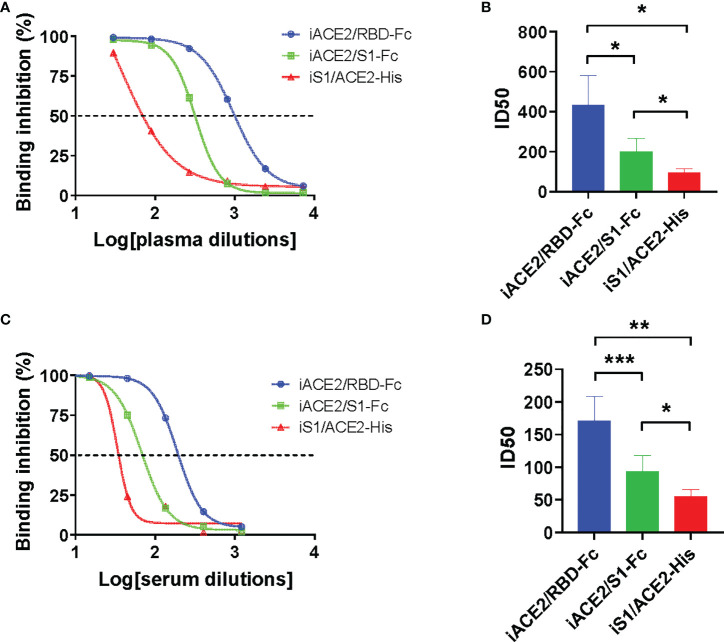
Comparison of ID50 values determined by models of iACE2/RBD-Fc, iACE2/S1-Fc and iS1/ACE2-His. Three representative sample dilution response inhibition curves of a convalescent plasma sample and a vaccine serum sample determined by the models of iACE2/BD-Fc, iACE2/S1-Fc and the iS1/ACE2-His were plotted and shown in **(A, C)**, respectively. The ID50 values of 12 convalescent plasma samples and 18 vaccine serum samples determined by the three models were compared and shown in **(B, D)**, respectively. The 18 vaccine serum samples were randomly selected from the samples collected after two weeks post the 2nd dose of immunization. P<0.05, 0.01 and 0.001 are represented as *, ** and ***, respectively.

The detection sensitivity of models iACE2/RBD-Fc, iACE2/S1-Fc and iS1/ACE2-His in detecting SARS-CoV-2 nAbs was further evaluated using 12 COVID-19 convalescent plasma samples and 18 SARS-CoV-2 inactivated vaccine serum samples. The neutralization activities, represented as ID50 values, of the same sample determined by three models were compared. As shown in **Figure 3**, iACE2/RBD-Fc model-determined ID50 values were significantly greater than that determined by iACE2/S1-Fc (**Figure 3A**) and iS1/ACE2-His (**Figure 3B**) for both convalescent patient plasma samples and vaccine serum samples (**Figures 3C, D**), demonstrating that iACE2/RBD-Fc model exhibited the highest sensitivity. Thus, iACE2/RBD-Fc model was focused in the following studies.

### Further assessment of iACE2/RBD-Fc model

**Figure 4:**
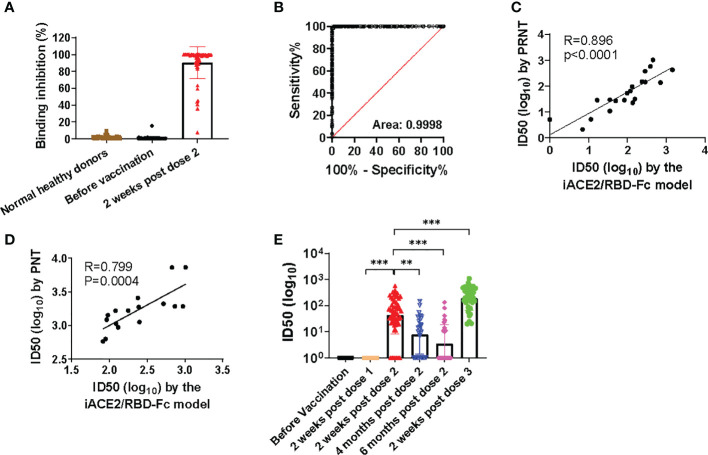
Assessment of sensitivity, specificity, accuracy and validation of iACE2/RBD-Fc model. To assess sensitivity and specificity of iACE2/RBD-Fc model, paired serum samples collected from 60 donors before the 1st dose of immunization and at two weeks post the 2nd dose of immunization were 5-fold diluted, then determined by iACE2/RBD-Fc model for activity to inhibit binding of the RBD-Fc on the ACE2 bead. The binding inhibition activity of each sample was shown in Panel **(A)** ROC curve analysis of the data shown in **(A)** was shown in **(B)**. To assess accuracy of iACE2/RBD-Fc model, ID50 values of 12 convalescent plasma samples and 8 vaccine serum samples were determined by the PRNT and the iACE2/RBD-Fc model, and ID50 values of 16 convalescent plasma samples were determined by the PNT and the iACE2/RBD-Fc model. The correlation of ID50 values determined by two assays of the PRNT/the iACE2/RBD-Fc model and two assays of the PNT/the iACE2/RBD-Fc model was shown in **(C, D)**, respectively. ID50 values of serum samples collected from the SARS-CoV-2 vaccine recipients at different time points during the vaccination course were determined by iACE2/RBD-Fc model and shown with its geometric mean in Panel **(E)** P<0.01 and 0.001 are represented as ** and ***, respectively.

The sensitivity and specificity of iACE2/RBD-Fc model in detecting nAb was further evaluated using sera of the same vaccine recipients collected before immunization and after two weeks post the 2^nd^ dose of immunization with a SARS-CoV-2 vaccine as well as the normal healthy donors collected in Taiyuan city. As shown in **Figure 4A**, under a serum dilution fold at 1:5, 4 out of 104 normal healthy donors and 1 out of 60 samples collected before immunization showed weak activities to inhibit binding between RBD-Fc and ACE2 bead. In contrast, >90% binding inhibition were detected in most samples collected after two weeks post the 2^nd^ dose of immunization. The sensitivity and specificity of iACE2/RBD-Fc model to discriminate between vaccinee recipients before and after immunization using binding inhibition rate as a parameter were analyzed by the ROC curve. The area under the curve was 0.9998 (**Figure 4B**). The ROC generated a cutoff binding inhibition rate of 6.59% using the ROC Youden J statistic, and the cutoff resulted in a specificity of 98.77% at a sensitivity of 100%. Furthermore, the ID50 values determined by iACE2/RBD-Fc model were positively correlated with those determined by PRNT (**Figure 4C**) and PNT (**Figure 4D**). Upon iACE2/RBD-Fc model was employed to test the longitudinal samples collected from vaccine recipients enrolled in Shanxi University at different time points during the vaccination course, significant neutralizing activity was detected in most (88.3%) and all (100%) samples collected after the 2^nd^ and 3^rd^ dose of immunization, respectively, and geometric mean of IC50 values of the samples collected after two weeks post the 3^rd^ dose of immunization were 4.4 times greater than those of the sample collected after two weeks post the 2^nd^ dose of immunization (**Figure 4E**). In contrast, neutralizing activity was undetectable in the samples collected before and after the 1^st^ dose of immunization (**Figure 4E**). Time dependent drop of neutralizing activity was also observed in the samples collected at three time points post the 2^nd^ dose of immunization (**Figure 4E**). The nAb kinetics in the vaccine recipients determined by iACE2/RBD-Fc model was consistent with that determined by PNT as shown in a recent study (26). The results indicated that iACE2/RBD-Fc model can accurately detect the SARS-CoV-2 nAb produced in human body in high sensitivity and specificity, and is qualified for detection of nAb elicited by a SARS-CoV-2 in the real-world setting.

### Comparison of detection sensitivity between iACE2/RBD-Fc model and a commercial sVNT ELISA kit

**Figure 5:**
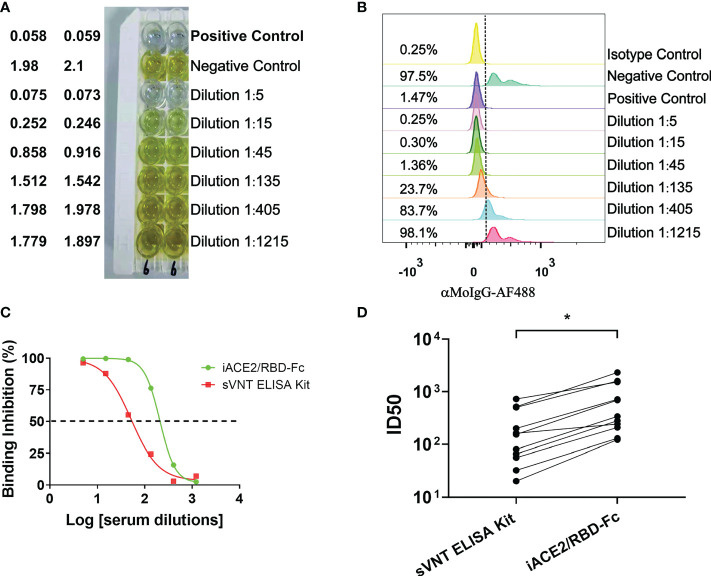
Comparison of ID50 values determined by the sVNT ELISA kit and iACE2/RBD-Fc model. ID50 values of 7 vaccine serum samples and 4 convalescent plasma samples were determined by the commercial ELISA based sVNT kit and the iACE2/RBD-Fc model. A panel of raw data for determination of neutralization activity of one vaccine serum by the sVNT ELISA kit and the iACE2/RBD-Fc model was shown in **(A, B)**, respectively. Two sample dilution response inhibition curves generated from the data shown in Panel **(A, B)** were plotted and shown in **(C)**. ID50 values of 11 samples determined by the two assays were compared and shown in **(D)**. The dash line was used to indicated the serum dilution fold with an activity to inhibit 50% binding of the RBD-Fc on the ACE2 bead. p value <0.05 is represented as *.

A commercial sVNT ELISA kit (GenScript cPass SARS-CoV-2 neutralization Antibody detection kit, GenScript, China) was produced according to the method invented by Tan, et al. (14), in which the ACE2 protein is immobilized and the HRP-conjugated spike RBD protein is soluble, resembling the iACE2/RBD-Fc model. The ID50 values of vaccine serum samples and COVID-19 convalescent plasma samples were determined by iACE2/RBD-Fc model and commercial sVNT ELISA kit in parallel. Results showed that the ID50 values determined by the iACE2/RBD-Fc model were significantly greater by approximate 3.7 times than that determined by the sVNT ELISA kit (**Figures 5A–D**), indicating that the sensitivity of iACE2/RBD-Fc model is superior to that of sVNT ELISA kit in detecting SARS-CoV-2 nAb.

### Determination of mechanism underlying a high sensitivity of iACE2/RBD-Fc model in detection of SARS-CoV-2 nAb

**Figure 6:**
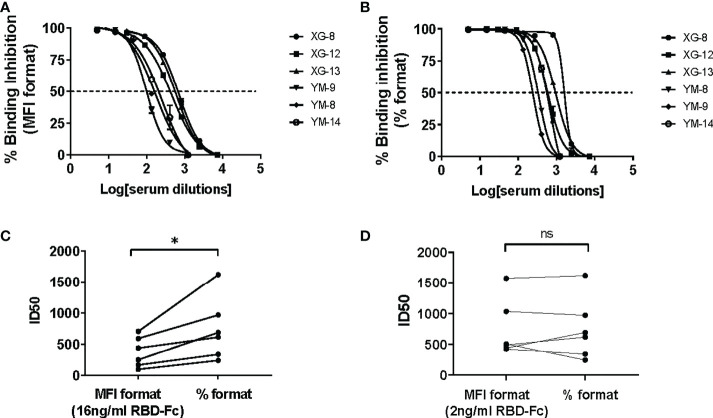
Comparison of ID50 values determined by the % formatted and the MFI formatted iACE2/RBD-Fc models. The sample dilution response inhibition curves of 3 convalescent plasma samples and 3 vaccine serum samples determined by the % formatted iACE2/RBD-Fc model using 2 ng/ml of RBD-Fc and the MFI formatted iACE2/RBD-Fc model using 16 ng/ml of RBD-Fc were shown in **(A, B)**, respectively. The ID50 values of the 6 samples determined by the % and MFI formatted iACE2/RBD-Fc models were compared and shown in **(C)**. The ID50 values of the 6 samples determined by the % formatted iACE2/RBD-Fc model and the MFI formatted iACE2/RBD-Fc model using 2 ng/ml of RBD-Fc for each assay were compared and shown in **(D)**. P values <0.05 and ≥0.05 are represented as * and ns, respectively.

In contrast to the CB based ELISA, CLIA or luminex SARS-CoV-2 nAb assay that determined the efficacy of binding inhibition between the soluble protein and its immobilized counterpart protein using colorimetric or chemiluminescent density in reaction solution (for the ELISA or CLIA assay) or using fluorescent intensity (MFI) on beads (for the Luminex assay) as a parameter (14, 16, 18, 19), the BFNT models developed in this study measure efficacy of binding inhibition between the soluble protein and its counterpart protein-immobilized bead using percentage of the soluble protein bound beads as a parameter (% formatted assay). To evaluate the effect of these two different measurements on the sensitivity of iACE2/RBD-Fc model in detecting nAb, we developed a MFI-formatted iACE2/RBD-Fc assay. To determine an optimal concentration of the soluble RBD-Fc in the MFI formatted iACE2/RBD-Fc model, a dose dependent MFI of RBD-Fc on the whole ACE2 beads were measured and 16 ng/ml concentration was determined as an upper limit within the linear range of MFI (**Supplementary Figures 3A, B**). Therefore, the diluted sample was pretreated with 16 ng/ml RBD-Fc in the MFI formatted iACE2/RBD-Fc model instead of 2 ng/ml RBD-Fc used in the % formatted iACE2/sRBD-Fc model. As shown in **Figures 6A–C**, the ID50 values of 3 vaccinated serum samples and 3 convalescent plasma samples determined by % formatted iACE2/RBD-Fc model were significantly increased by 2.1 folds than those determined by the MFI formatted iACE2/RBD-Fc model. However, upon reducing the soluble RBD-Fc to 2 ng/ml in the MFI formatted iACE2/RBD-Fc model, the ID50 values of these samples determined by these two formats of iACE2/RBD-Fc model became comparable (**Figure 6D**). The results indicated that the lower concentration of soluble RBD-Fc accounted for the improved sensitivity of the % formatted iACE2/RBD-Fc model.

### Versatility assessment of the iACE2/RBD-Fc model

**Figure 7:**
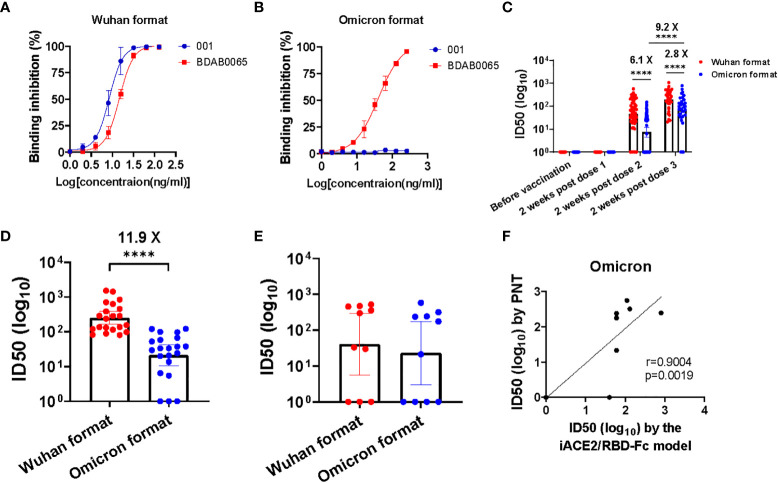
Assessment of the iACE2/RBD-Fc model in detection of omicron variant specific nAb. Detection of one SARS-CoV-2 Wuhan strain specific nAb 001 and one SARS-CoV-2 broadly nAb BDAB0065 by the iACE2/RBD-Fc model was shown in **(A)** (the format with Wuhan strain RBD-Fc, in short as Wuhan format) and **(B)** (the format with omicron BA,1 variant RBD-Fc, in short as omicron format). ID50 values of serum samples collected from the SARS-CoV-2 vaccine recipients at time points of before vaccination (n=60), 2 weeks post the first dose (n=60), 2 weeks post the second dose (n=60) and 2 weeks post the third dose (n=34) during the vaccination course were determined by the Wuhan format and the omicron format of iACE2/RBD-Fc model and were shown in **(C)**. The Wuhan format and the omicron format of iACE2/RBD-Fc model also were employed to detect plasma samples from 21 convalescent patients previously infected by SARS-CoV-2 Wuhan strain and10 patients breakthrough-infected by SARS-CoV-2 omicron BA.1 variant, the determined ID50 values of each group were shown in **(D, E)**, respectively. To assess the accuracy of the omicron format of iACE2/RBD-Fc model, the ID50 values of 10 breakthrough infection plasma samples (the identical samples shown in Panel **E**) were determined by PNT using omicron BA.1 variant pseudovirus. Correlation of ID50 values of the 10 samples determined by the PNT using omicron pseudovirus and the omicron format of iACE2/RBD-Fc model was shown in **(F)**. In the **(C–E)**, geometric mean and 95% confidence interval of ID50 values of the indicated group samples was shown. P values <0.0001 is represented as ****.

To evaluate efficacy of the iACE2/RBD-Fc model in detecting omicron variant specific nAb, the prototype iACE2/RBD-Fc model was modified by replacement of the Wuhan strain RBD-Fc with the omicron BA.1 variant RBD-Fc, then the developed omicron variant format of iACE2/RBD-Fc model (in short as omicron format) was assessed to detect SARS-CoV-2 specific monoclonal nAbs, vaccine serum samples, convalescent plasma samples and omicron variant breakthrough infection samples with its prototype Wuhan strain format of iACE2/RBD-Fc model (in short as Wuhan format) as a control. As shown in **Figures 7A, B**, the Wuhan format detected both the Wuhan strain specific nAb 001 and the broadly nAb BDAB0065, whereas the omicron format merely detected the broadly nAb BDAB0065. Upon detection of vaccine serum samples, the omicron format did not detect any neutralizing activity in all samples collected before vaccination and at 2 weeks post the first dose of vaccination and apparent neutralization activity until the third dose post vaccination (geometric mean neutralization titer ID50, GMT50, is 67.2) (**Figure 7C**). Consistent with the low levels of neutralization activity detected by the omicron format in the samples collected at 2 weeks post the second dose of vaccination (GMT50 = 7.3), the omicron format detected neutralization activity in the convalescent samples was as low as GMT50 = 21.2, which was 11.9 times less than that detected by the Wuhan format (GMT50 = 251.3) (**Figure 7D**). On the other hand, neutralization activity of the breakthrough infection plasma samples detected by the omicron format and the Wuhan format were comparable (**Figure 7E**). The neutralization activity in samples of vaccine serum, convalescent plasma and breakthrough infection plasma profiled by the omicron format of iACE2/RBD-Fc model is consistent with those profiled by the conventional PNT and PRNT using omicron variant pseudovirus or live virus (27–29). Furthermore, there was a significant correlation between the omicron specific nAb activity determined by the PNT and the omicron format of iACE2/RBD-Fc model (**Figure 7F**). These data indicated that the omicron format of iACE2/RBD-Fc could efficiently detect omicron specific neutralization activity with high specificity.

## Discussion

In this study, we developed a highly sensitive and specific assay to detect SARS-CoV-2 nAb by comparing multiple competitive binding models side by side, and found that the tag of soluble ACE2, type of spike proteins, and methods to calculate binding rate between ACE2 and spike protein significantly affected the detection performance of the assay models.

We found that Fc tag in the soluble proteins may have different effects on the detection of SARS-CoV-2 nAb by the models we established. For the models using immobilized RBD or S1 protein and soluble ACE2 (iRBD/ACE2-Fc, iS1/ACE2-Fc, iRBD/ACE2-His and iS1/ACE2-His), the presence of Fc tag, rather than His tag, in the soluble ACE2 profoundly decreased the sensitivity in detecting SARS-CoV-2 neutralizing mAb 001 (**Figures 2C–F**) or abolished the ability of iRBD/ACE2-Fc and iS1/ACE2-Fc models to detect SARS-CoV-2 nAb in COVID-19 covalescent plasma samples and vaccine serum samples (data not shown). In contrast, for the models using the immobilized ACE2 and soluble RDB or S1 protein (iACE2/RBD-Fc and iACE2/S1-Fc), the presence of Fc tag in the soluble RBD or S1 protein didn’t affect nAb detection and even could improve the efficiency of interaction between the soluble spike protein and the immobilized ACE2 (**Supplementary Figures 2A–D**, panel A vs panel C; panel B vs panel D). ACE2 was physiologically expressed as a homodimer on the cell membrane through its neck domain localized in the C terminus of ACE2 extracellular domain, and the ACE2 homodimer might bind to two trimeric spike proteins through the RBD during SARS-CoV-2 infection (30, 31). The ACE2-Fc and ACE2-His proteins are produced by fusion of the ACE2 extracellular domain with a mouse Fc tag or His tag, so these two proteins are both homodimer proteins. Although the immobilized monomeric S1 or RBD protein was observed to be able to detect SARS-CoV-2 nAb upon combination with the ACE2-His protein, they lost such the ability upon combination with the ACE2-Fc protein, suggesting a distinct structural interaction which may be hindered by the presence of Fc tag. On the other hand, the soluble Fc-tagged ACE2 was reported to efficiently detect SARS-CoV-2 nAb upon combination with the immobilized trimeric full spike protein (16). These data implied that both the type of spike proteins and the tag of ACE2 protein affected the sensitivity of the assays using immobilized spike protein and soluble ACE2 protein.

The RBD is a small middle part of the S1 subunit. The model using soluble His-tagged dimeric ACE2 and immobilized monomeric RBD protein (iRBD/ACE2-His), rather than S1 protein (iS1/ACE2-His) detected pseudo neutralization activity for two SARS-CoV-2 non-neutralizing mAbs (especially for mAb CR3022) (**Figures 2E, F**), suggesting that direct immobilization of RBD protein to the bead might exert unexpected conformational effect for a smaller protein, thus making itself over sensitive to the RBD non-neutralizing antibody. These data hinted the requirement of assessing the specificity of CB-based SARS-CoV-2 nAb assays in rigorous ways to exclude the non-specific cross neutralization activity for precise detection of SARS-CoV neutralizing nAb (14).

Given there were more neutralizing epitopes on the S1 subunit than the RBD (32), the iACE2/S1-Fc model was supposed to be more sensitive than the iACE2/RBD-Fc model in detection of SARS-CoV 2 nAb. Unexpectedly, a better sensitivity was found for the iACE2/RBD-Fc model (**Figure 3**), which was consistent with the observation by Tan et al. using the sVNT ELISA assay (14). This observation indicates that blocking interaction between ACE2 and RBD of spike protein might represent the major mechanism behind the detection of SARS-CoV-2 nAb by the current CB-based SARS-CoV-2 nAb assays.

Among the three models capable of detecting nAb in high specificity (iACE2/RBD-Fc, iACE2/S1-Fc and iS1/ACE2-His), an inverse correlation between the detection sensitivity and the optimal binding concentrations of soluble protein used was observed (**Supplementary Figures 2A, B, F** and **Figure 3**). The lower optimal binding concentration of RBD-Fc in the % formatted iACE2/RBD-Fc assay than the MFI formatted iACE2/RBD-Fc assay accounted for a better sensitivity of the former than the latter in detecting SARS-CoV-2 nAb (**Figures 6C, D**). These results demonstrated that a low optimal binding concentration of the soluble protein was one of the keys to achieving a high sensitivity of the CB-based SARS-CoV-2 nAb assays, and measuring the percentage of soluble protein bound bead as a parameter to calculate the rate of binding inhibition between soluble protein and its counterpart protein-immobilized bead is an effective way to lower the optimal binding concentration of the soluble protein. In line with the finding of this study, our group previously developed a % formatted CB-based assay to detect activity of immune checkpoint inhibitors, which is more sensitive than the CB-based ELISA or SPR based assays [26]. Therefore, the % formatted CB assay might be a promising strategy to detect in high sensitivity various kinds of antibodies with blocking activity.

The sVNT assay represents the first CB-based SARS-CoV-2 nAb assay and has been widely used for diagnostic detection of SARS-CoV-2 natural infection and evaluation of SARS-CoV-2 vaccine efficacy (33–35). While the sensitivity and specificity of sVNT assay have been confirmed in detection of SARS-CoV-2 nAb [14], the commercial sVNT kit still showed insufficient sensitivity to detect early sera samples post symptom onset in diagnosis of acute SARS-CoV-2 infection [27,28]. This flaw may be secured by the study of this report since the iACE2/RBD-Fc model established here showed 3.7 times more sensitivity in detection of SARS-CoV-2 nAb than the commercial sVNT kit (**Figures 5A–D**). The optimal binding concentration of RBD-HRP in the sVNT ELISA kit assay is 30 ng/ml (14), which is 15 times more than that of RBD-Fc in the iACE2/RBD-Fc model assay. The much lower concentration of the soluble RBD protein in the iACE2/RBD-Fc model assay than the commercial sVNT kit assay might account for the better sensitivity of the former.

Consistence with other reported CB based SARS-CoV-2 nAb assays, the iACE2/RBD-Fc assay developed in this study can detect nAb against both the prototype SARS-CoV-2 strain and its emerging variants in a biosafety hood under BL1 facility with a turn-around time less than 2 hrs (16, 19), The high-throughput is feasible upon detection by a flow cytometry equipped with a high-throughput sampling system or a luminex instrument. On the other hand, the very small amount of the recombinant proteins (3.6 ng of ACE2-Avi and 0.2 ng of RBD-Fc) used in each test possesses the current iACE2/RBD-Fc assay of high sensitivity and high cost-effectiveness meanwhile, which is not reported previously. These merits make the iACE2/RBD-Fc assay a promising alternative assay of the conventional PNT and PRNT which are flawed in a long turnaround-time (2-7 days), requirement of an intermediate or high biosafety level facility (BL-2 or BL-3 lab), high cost for associated reagent/labor and low standardization due to high variability in cell types, cell numbers and input of virus number.

In summary, the results in this report provided a valuable novel method to detect SARS-CoV-2 nAb in high sensitivity, and elucidated deeply the working principles of the CB-based SARS-CoV-2 nAb assays, which will benefit the vaccine evaluation and earlier diagnosis of SARS-CoV-2 and even other viruses.

## Data availability statement

The original contributions presented in the study are included in the article/**Supplementary Material**. Further inquiries can be directed to the corresponding authors.

## Ethics statement

The studies involving human participants were reviewed and approved by Ethics Committee of Shanxi University, Shanxi University; Ethics Committee of Taiyuan blood center, Taiyuan blood center; Ethics Committee of the First Affiliated Hospital of USTC, the First Affiliated Hospital of USTC. The patients/participants provided their written informed consent to participate in this study.

## Author contributions

Project administration, conceptualization and original drafting: QLM. Design, funding acquisition and supervision: QLM, CW, and YG. Investigation, data analysis and curation: XY, ZZ, and QMM. Investigation: SL, YL, JW, and XW. Critical revision of the article: LX and BX. Resource: DZ and BY. All authors contributed to the article and approved the submitted version.

## Funding

This work was supported by the Shanxi Province Education Department under grant: Transformation of Scientific and Technological Achievements Programs of Higher Education Institutions in Shanxi (TSTAP); the Innovation Program of graduate in Shanxi under grant 2021Y133; the Programme of Introducing Talents of Discipline to Universities under grant D21004; the National Natural Science Foundation of China under grant 32100133; the Key R&D Plan of Shanxi Province under Grant 202003D31006/GZ; the Shanxi “1331 Project” Collaborative Innovation Centre, 1331 CIC under grant 206541001.

## Acknowledgments

We thank all participants of this study and Dr. Donald. D. Anthony and Dr. Huiguang Li for helpful comments on preparation of the manuscript.

## Conflict of interest

The authors declare that the research was conducted in the absence of any commercial or financial relationships that could be construed as a potential conflict of interest.

## Publisher’s note

All claims expressed in this article are solely those of the authors and do not necessarily represent those of their affiliated organizations, or those of the publisher, the editors and the reviewers. Any product that may be evaluated in this article, or claim that may be made by its manufacturer, is not guaranteed or endorsed by the publisher.
