# Supplementary material for: A highly sensitive bead-based flow cytometric competitive binding assay to detect SARS-CoV-2 neutralizing antibody activity
Source: Front Immunol. 2022 Nov 30;13:1041860. doi: 10.3389/fimmu.2022.1041860 (PMC9748424; doi:10.3389/fimmu.2022.1041860)
Supplement: Supplementary file 1 [file DataSheet_1.docx]

**Supplemental figures**


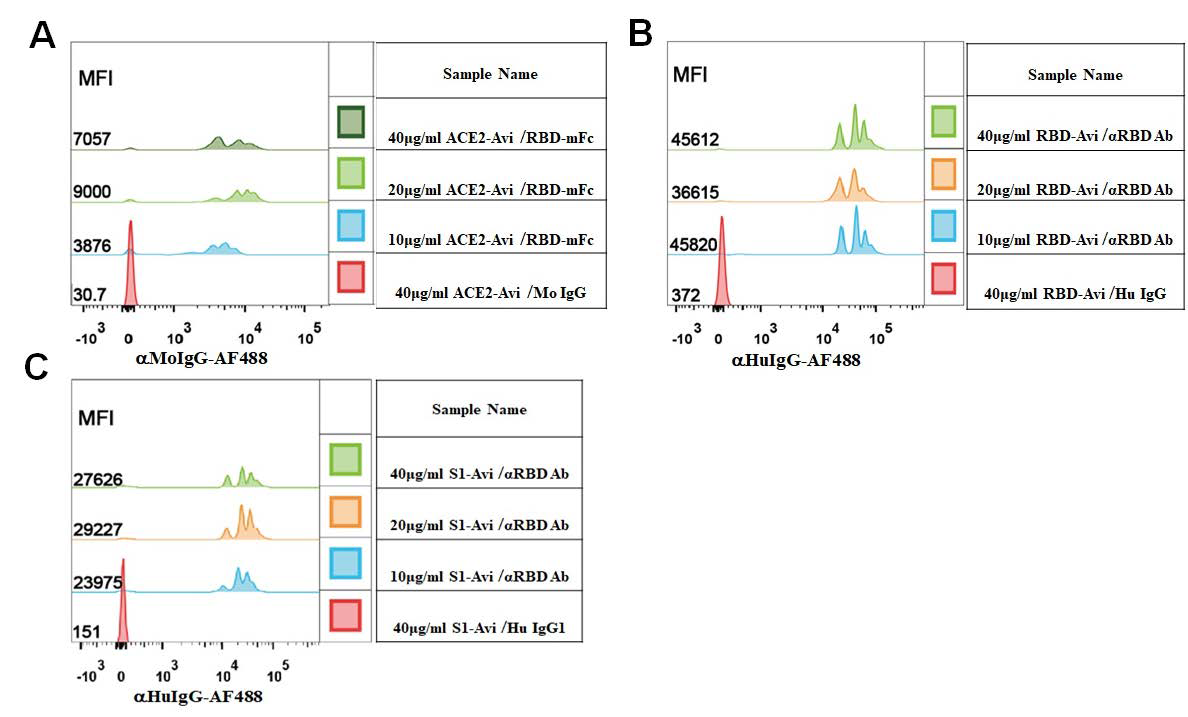


**S. Fig. 1. Titration of ACE2-Avi, RBD-Avi and S1-Avi for saturation immobilization on the SA bead.** The SA bead was immobilized with ACE2-Avi, RBD-Avi or S1-Avi at concentrations of 10 μg/ml, 20 μg/ml and 40 μg/ml to generate three stocks of ACE2 bead, RBD bead or S1 bead. 1 μl of each stock of ACE2 bead were twice washed and incubated with 1 μg/ml of RBD-Fc at RT for 30 mins with one additional incubation of ACE2 bead (preparation with 40 μg/ml of ACE2-Avi) with 1 μg/ml mouse IgG1 as a negative control. The pre-treated ACE2 bead samples were washed and stained by 0.5 μg/ml of AF488 conjugated goat anti mouse IgG polyclonal antibody (pAb), followed by washing and flow cytometry analysis. One representative staining result was shown in (**A**). 1 μl of each stock of RBD bead or S1 bead were twice washed and incubated with 0.1 μg/ml of humanized anti RBD mAb (HMB001-N) at RT for 20 mins with one additional incubation of RBD bead or S1 bead with 0.1 μg/ml of human IgG as a negative control. The pre-treated RBD bead or S1 bead was washed and stained by 0.5 μg/ml of AF488 conjugated goat anti human IgG pAb, followed by washing and flow cytometry analysis. One representative result of staining RBD bead (**B**) and S1 bead (**C**) were shown.





**S. Fig2. Binding of ACE2 bead, RBD bead or S1 bead with their counterpart proteins.** 1 μl of ACE2 bead stock was incubated with 0.5, 1, 1.5, 2, 3 or 4 ng/ml of RBD-Fc (Wuhan strain) or incubated with 1, 2, 4, 8, 16, 32 or 64 ng/ml of S1-Fc at RT for 30 mins, then washed and stained with 0.5 μg/ml of AF488 conjugated goat anti mouse IgG pAb. After twice washing, the RBD-Fc (Wuhan strain) incubated ACE2 bead samples and the S1-Fc incubated ACE2 bead samples were subjected to flow cytometry analysis. The percent RBD-Fc (Wuhan strain) bound ACE2 bead versus various concentrations of RBD-Fc (Wuhan strain) and the percent S1-Fc bound ACE2 bead versus various concentrations of S1-Fc were shown in **(A)** and (**B)**, respectively. 1 μl of RBD bead stock was incubated with 0.5, 1, 2, 4, 8, 16 or 32 ng/ml of ACE2-Fc at RT for 30 min; 1 μl of S1 bead stock was incubated with 1, 2, 4, 8, 16, 32, 64 or 128 ng/ml of ACE2-Fc at RT for 30 min, then the ACE2-Fc pre-incubated RBD bead or S1 bead samples were washed and stained with 0.5 μg/ml of AF488 conjugated goat anti mouse IgG pAb at RT for 20 mins. After twice washing, the stained RBD or S1 bead samples were subjected to flow cytometry analysis. The percent ACE2-Fc bound RBD bead or S1 bead versus various concentrations of ACE2-Fc were shown in **(C)** and (**D)**, respectively. 1 μl of RBD bead stock or S1 bead stock was incubated with 25, 50, 100, 200, 400 or 800 ng/ml of ACE2-His at RT for 30 min, then washed and stained with 0.12 μg/ml of PE conjugated mouse anti His tag mAb at RT for 20 min, followed by washing and flow cytometry analysis. The percent ACE2-his bound RBD bead or S1 bead versus various concentrations of ACE2-His were shown in **(E)** and (**F)**, respectively. 1 μl of ACE2 bead stock was incubated with 0.5, 1, 1.5, 2, 3 or 4 ng/ml of RBD-Fc (omicron variant) at RT for 30 mins, then washed and stained with 0.5 μg/ml of AF488 conjugated goat anti mouse IgG pAb. After twice washing, the RBD-Fc (omicron variant) incubated ACE2 bead samples were subjected to flow cytometry analysis. The percent RBD-Fc (omicron variant) bound ACE2 bead versus various concentrations of RBD-Fc (omicron variant) were shown in **(G)**.


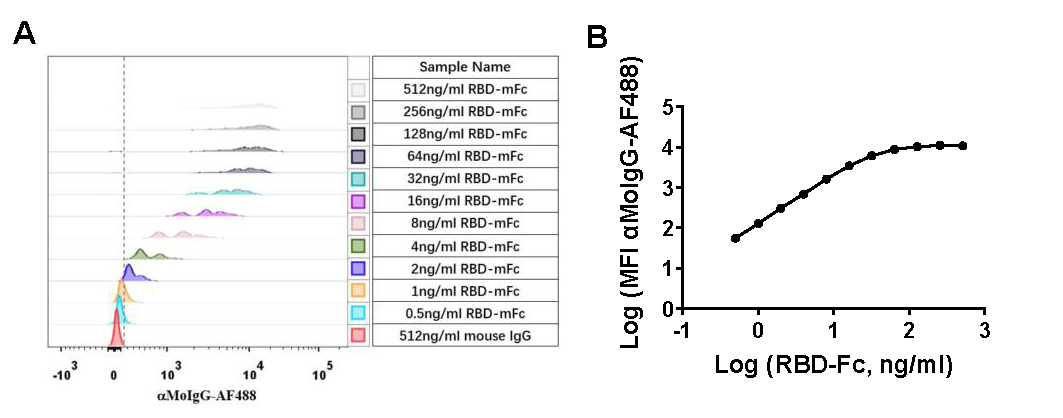


**S. Fig3. MFI analysis of binding of RBD-Fc on ACE2 bead.** 1μl of ACE2 bead stock was incubated with 0.5, 1, 2, 4, 8, 16, 32, 64, 128, 256 and 512 ng/ml of RBD-Fc at RT for 30 mins, then washed and stained with 0.5 μg/ml AF488 conjugated goat anti mouse IgG pAb. After twice washing, the RBD-Fc incubated ACE2 bead samples were subjected to flow cytometry analysis. One representative staining result of the RBD-Fc incubated ACE2 bead samples were shown in **(A)**. The data of MFI of RBD-Fc on the whole ACE2 bead versus various concentrations of RBD-Fc were plotted and shown in **(B)**.
